# Study of Precipitated Secondary Phase at 700 °C on the Electrochemical Properties of Super Duplex Stainless Steel AISI2507: Advanced High-Temperature Safety of a Lithium-Ion Battery Case

**DOI:** 10.3390/ma17092009

**Published:** 2024-04-25

**Authors:** Byung-Hyun Shin, Seongjun Kim, Jinyong Park, Jung-Woo Ok, Dohyung Kim, Jang-Hee Yoon

**Affiliations:** 1 Busan Center, Korea Basic Science Institute, Busan 46742, Republic of Korea; lemonhouse211@kbsi.re.kr (B.-H.S.); seongjunk@kbsi.re.kr (S.K.); jinyongp@kbsi.re.kr (J.P.); jwok@kbsi.re.kr (J.-W.O.); 2 Innovative Graduate Education Program for Global High-Tech Materials and Parts, Pusan National University, Busan 46241, Republic of Korea

**Keywords:** super duplex stainless steel, microstructure at 700 °C, electrochemical behavior, Li-ion battery case material, precipitation of secondary phase

## Abstract

Super duplex stainless steel (SDSS) is a suitable structural material for various engineering applications due to its outstanding strength and corrosion resistance. In particular, its high-temperature strength can enhance the safety of electronic products and cars. SDSS AISI2507, known for its excellent strength and high corrosion resistance, was analyzed for its microstructure and electrochemical behavior at the ignition temperature of Li-ion batteries, 700 °C. At 700 °C, AISI2507 exhibited secondary phase precipitation values of 1% and 8% after 5 and 10 h, respectively. Secondary phase precipitation was initiated by the expansion of austenite, forming sigma, chi, and CrN phases. The electrochemical behavior varied with the fraction of secondary phases. Secondary phase precipitation reduced the potential (From −0.25 V to −0.31 V) and increased the current density (From 8 × 10^−6^ A/cm^2^ to 3 × 10^−6^ A/cm^2^) owing to galvanic corrosion by sigma and chi. As the fraction of secondary phases increased (From 0.0% to 8.1%), the open circuit potential decreased (From −0.25 V to −0.32 V). Secondary phase precipitation is a crucial factor in reducing the corrosion resistance of SDSS AISI2507 and occurs after 1 h of exposure at 700 °C.

## 1. Introduction

With the increasing demand for portable electronic devices and electric vehicles, the demand for lithium-ion batteries (Li-ion batteries), which serve as energy storage devices, is increasing annually [1,2,3]. Li-ion batteries exhibit variations in heat generation depending on their usage conditions, thus they require high safety standards [4,5,6]. Recently, explosions involving portable electronic devices have become more frequent, posing risks, particularly when subjected to high-temperature heating, such as in electric vehicles. During thermal runaway reactions, battery temperatures can reach as high as 700 °C [7,8,9]. Many studies have been conducted on numerous catalysts and systems to control the exothermic reactions they cause [7,8,10,11]. Consequently, battery casing materials derived from stainless steel AISI304 (Melting point: 1400 °C) have replaced materials with low melting temperatures like aluminum (melting temperature: 670 °C), thus enhancing safety standards.

Stainless steel AISI304, a member of the austenite stainless steel series, is one of the most widely used stainless steel [4,12,13]. Stainless steels are classified into austenite, ferrite, martensite, and duplex series based on their main phases [14,15,16]. Austenite stainless steel exhibits excellent corrosion resistance [17,18,19]. However, ferrite and martensite stainless steels possess inferior corrosion resistance, superior strength, and high-temperature strength. Duplex stainless steel exhibits excellent corrosion resistance and strength [20,21,22]. Owing to the differences in their performance, the stainless steel types are classified based on their intended application. Therefore, to ensure stability at temperatures as high as 700 °C in Li-ion batteries, materials with both corrosion resistance and high temperature resistance should be utilized.

Duplex stainless steel, which comprises both austenite and ferrite phases, offers excellent strength, corrosion resistance, and high-temperature strength, which contribute to the enhanced safety of Li-ion battery casings, even during heat generation. Duplex stainless steel is graded according to the pitting resistance equivalent number (PREN) [14,23]. Super duplex stainless steel (SDSS), with a PREN exceeding 40, exhibits superior strength and corrosion resistance, making it suitable for safety enhancement. AISI2507, with a composition of 25 wt% Cr and 7 wt% Ni, exhibits a PREN of 42. AISI2507; it was originally developed for marine applications, is currently being studied in various fields to maximize its excellent properties. However, research on Li-ion battery casings is limited.

Research on AISI2507 is essential for ensuring the safety of Li-ion batteries due to its strength (780 MPa), high-temperature strength (300 MPa at 700 °C), corrosion resistance (50 years in sea water), and low maintenance cost. Previous studies have explored various aspects such as heat treatment and secondary phase precipitation. Nillson investigated changes in the phase fractions and corrosion resistance of AISI2507 with heat treatment temperature, providing foundational data for several SDSS studies [14,24]. Martins examined secondary-phase precipitation after heat treatment at 920 °C [25]. Fande researched two types of welding involving SDSS and austenitic stainless steel and revealed trends in SDSS resulting from heat treatment during welding [26]. This shift in SDSS research indicates an increase in machining research, which facilitates its utilization. However, research on the electrochemical properties of AISI2507 at 700 °C and how they can enhance the safety of battery casings remains absent.

To enhance the safety of Li-ion batteries, AISI2507 was applied as the material for the Li-ion battery case, and the precipitation behavior of secondary phases was analyzed to assess its high-temperature safety through its microstructure and electrochemical behavior. This study analyzes the microstructure changes and electrochemical behavior of AISI2507 at 700 °C to enhance the safety of Li-ion batteries. AISI2507 was subjected to heat treatment at 700 °C (The ignition temperature of batteries) for different durations (0, 1, 5, and 10 h). The resulting microstructure changes were analyzed using open circuit potential (OCP), electrochemical impedance spectroscopy (EIS), and potentiodynamic polarization tests.

## 2. Experimental Method

### 2.1. Materials

The composition of the cast AISI2507 used in this study was analyzed using inductively coupled plasma mass spectrometry (ICP-MS; Thermo Fisher Scientific, Waltham, MA, USA). The chemical composition is presented in Table 1 [20,27,28]. AISI2507 contained 25 wt% Cr, and 7 wt% N as the major constituents, with a PREN of 42, as calculated using Equation (1).

The major alloying elements, Cr, Mo, and N, play a crucial role in reinforcing the passive layer of AISI2507 (Cr_2_O_3_) [26,29]. Cr and Mo stabilize the ferrite phase owing to their body-centered cubic (BCC) structures, thus promoting the formation of a passive layer. Conversely, Ni and Mn, with their face-centered cubic (FCC) structures, contribute to the stabilization of the austenite phase [17,30,31].

Pitting resistance equivalent (PRE) = wt% Cr + 3.3 wt% Mo + 16 wt% N(1)

The samples used for the analysis were cast in the form of round bars with a diameter of 50 mm and a length of 100 mm. Subsequently, the samples were machined at 10 mm intervals and used to analyze the electrochemical behavior after heat treatment.

### 2.2. Heat Treatment

**Figure 1:**
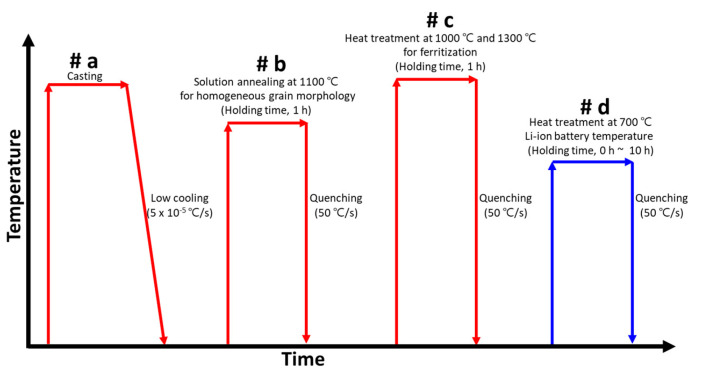
Schematic of heat treatment conditions to observe microstructural changes in super duplex stainless steel AISI2507 at the ignition temperature (700 °C) of Li-ion batteries (Red line is manufacturing process, and blue line is aging temperature of Li-ion batteries at ignition temperature).

The heat treatment of AISI2507 at 700 °C was performed in a box furnace. The dimensions of the box furnace were 300 mm × 300 mm × 300 mm, and the specimens were inserted into the furnace after they reached the target temperature of 700 °C. A schematic of the heat treatment conditions is shown in Figure 1.

The as-cast AISI2507 was air-cooled (#a); however, it exhibited precipitation, which led to cracking during subsequent processing. Solution annealing (#b) was performed to stabilize the composition and microstructure of AISI2507. The solution annealing of AISI2507 involved heating to 1100 °C, where the volume fractions of austenite and ferrite became equal, followed by rapid cooling at a rate of 50 °C/s. Subsequently, to observe the microstructural changes at 700 °C, the specimens were further subjected to ferritization annealing by heating to 1300 °C for 1 h and then rapidly cooling at 50 °C/s (#c). Subsequently, heat treatments were conducted at 700 °C for durations ranging from 0 to 10 h to (#d) examine both microstructure and electrochemical behavior.

### 2.3. Microstructure and Phase

The microstructural evolution with respect to heat treatment duration was analyzed using field-emission scanning electron microscopy (FE-SEM, SUPRA 40VP system, Zeiss, Oberkochen, Land Baden-Württemberg, Germany). Surface polishing with colloidal silica was performed to prepare the samples for microstructural examination, followed by electrolytic etching in a 10 wt% NaOH electrolyte solution under 5 V for 30 s [21,32,33]. The etched microstructures were observed and the volume fractions of the phases were determined according to ASTM E1245 [34].

The chemical composition and phase analyses were conducted using energy-dispersive spectroscopy (EDS, SUPRA 40VP system, Zeiss, Land Baden-Württemberg, Germany) and X-ray diffraction (XRD, D8 VENTURE, Stanford, CA, USA), respectively. The elucidation of the chemical composition by EDS analysis may not have accurately detected invasive elements such as nitrogen because of the small size of N atoms. The N chemical composition was calculated using the following formula:

N_r_ = chemical composition of N_Total_ wt% − Ferrite_VF_ × 0.05 wt% (2)

### 2.4. Electrochemical Behavior

The electrochemical properties of AISI2507 at 700 °C were analyzed over time using three techniques: open circuit potential (OCP), electrochemical impedance spectroscopy (EIS), and potentiodynamic polarization tests. The electrochemical analysis employed a three-electrode cell setup comprising working (Specimens), reference (Saturated calomel electrode), and counter electrodes. Two electrolyte solutions were used including a 3.5 wt% NaCl solution for OCP, EIS, and potentiodynamic polarization tests, following the ASTM G 61 test method [24,35,36].

The OCP measures potential changes with time. Although potential calculations are feasible for pure metals, they are not straightforward for alloys because of galvanic corrosion. In particular, stainless steel is susceptible to the influence of passive layers, making the OCP crucial for assessing material conditions. EIS was used to measure the resistance changes at frequencies ranging from 10^−2^ to 10^6^ Hz [37,38,39]. The surface layer composition can be determined by observing the changes in resistance with frequency and distribution. Potentiodynamic polarization testing was used to measure the changes in the current density with respect to the potential (Scan rate 0.167 mV) from −0.6 V to 1.2 V [40,41,42]. The potential indicates the onset of reactions, whereas the current density reflects the corrosion rate. This method is suitable for comparing corrosion behaviors under different material conditions.

## 3. Methods

### 3.1. Microstructure

**Figure 2:**
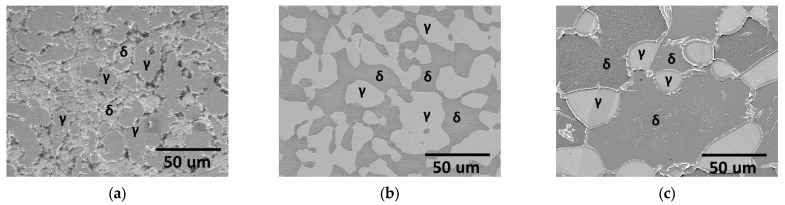
Microstructure (γ: austenite, δ: ferrite) of super duplex stainless steel AISI2507: (**a**) cast’s microstructure, (**b**) microstructure subjected to heat treatment at 1100 °C, and (**c**) microstructure subjected to heat treatment at 1300 °C.

The microstructural changes in AISI2507 after casting, annealing, and ferritization heat treatments were investigated (Figure 2) [14,20,27]. The microstructure of the cast AISI2507 (Figure 2a) appeared to be uneven and exhibited a fine austenitic morphology with a subtle presence of secondary phases. The microstructure annealed at 1100 °C (Figure 2b) exhibited an equal fraction of austenite and ferrite in a 5:5 ratio. According to the existing literature, annealing under such conditions with equal PREN values demonstrates optimal corrosion resistance. The ferritized microstructure (Figure 2c) exhibited a higher volume fraction of ferrite.

The volume fractions of the phase and chemical composition analysis results are tabulated in Table 2, along with the PREN calculations [14,22,28]. Differences in the major alloy compositions were observed depending on the material state and phase, which contributed to variations in the PREN. The cast AISI2507 austenite had lower chemical compositions of Cr and Mo than the heat-treated specimens. As the annealing temperature increased after casting, the volume fraction of ferrite increased, whereas the compositions of Cr and Mo in the ferrite decreased, leading to a decrease in the PREN [25,40,43]. Conversely, the chemical composition of the major alloys, excluding N in the austenite, increased, resulting in an increase in the PREN. Compared with the existing literature, the volume fractions of the phase and the chemical composition varied similarly with the heat treatment temperature, indicating the suitability of the heat treatment for the intended purposes [14,20,21].

**Figure 3:**
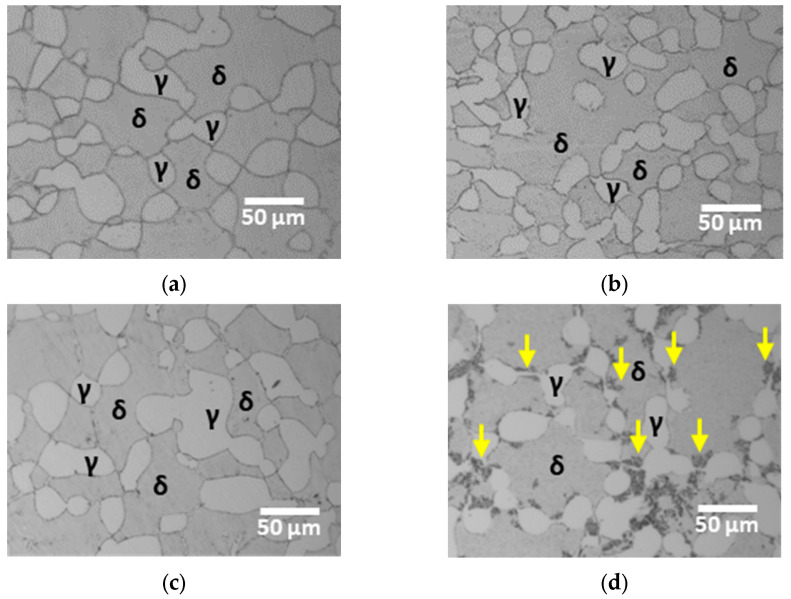
Microstructure (γ: austenite, δ: ferrite) of super duplex stainless steel AISI2507: (**a**) heat treatment for (**a**) 0 h at 700 °C; (**b**) heat treatment for 1 h at 700 °C; (**c**) heat treatment for 5 h at 700 °C; and (**d**) heat treatment for 10 h at 700 °C with secondary phases indicated by yellow arrows.

The post annealed microstructure exhibited a stable state characterized by uniform volume fractions of the phases and equal PREN differences [20,21,27]. Subsequent ferritization heat treatment resulted in decreased corrosion resistance, making the microstructural changes at 700 °C visible. The variations in the microstructure with the duration of heat treatment at 700 °C are shown in Figure 3. Subtle growth of the secondary phases was observed with increasing heat-treatment time [40]. These secondary phases were distinguished by their dark appearance owing to surface etching. The precipitation of secondary phases was observed with increasing heat treatment time at 700 °C.

**Figure 4:**
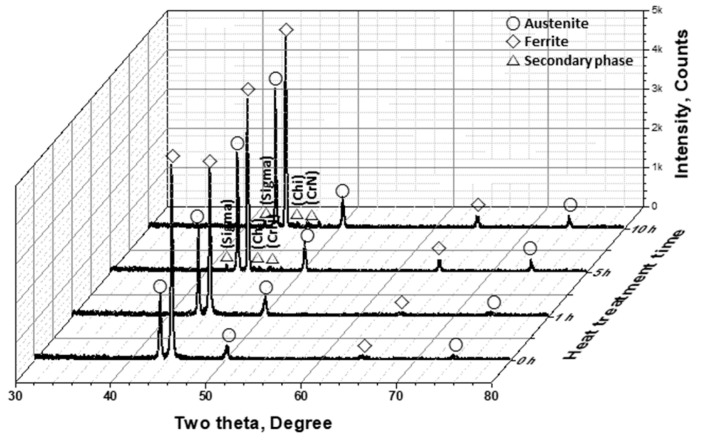
Two theta (degree) vs. intensity (counts) curves, X-ray diffraction pattern from 30° to 80° of super duplex stainless steel AISI2507.

Phase precipitation as a function of heat treatment duration was analyzed using XRD, (Figure 4) [25,40]. The secondary phases were identified as sigma, chi, and CrN, with the precipitation sites being located at the boundaries of the austenite phase. The secondary phase predominantly occurred in regions with higher austenite fractions. These findings suggest that the precipitation of secondary phases in the alloy was induced by the formation of precipitates owing to the growth of austenite.

The precipitation of the sigma phase was facilitated by the segregation of Cr and Mo at the boundaries of the austenite phase, resulting from the growth of austenite. The precipitated sigma phase, characterized by high levels of Cr and Mo within its composition, induced a Cr-depleted zone in the surrounding matrix, resulting in the precipitation of the chi phase. This sequential precipitation of secondary phases increased during the CrN formation owing to the inability of N to be incorporated into the sigma phase.

The precipitation mechanism of secondary phases is well understood. Precipitation occurred when the fraction of austenite exceeded 56% [14,24]. The precipitation of secondary phases is attributed to the transformation of ferrite to austenite rather than to the fraction of austenite. Therefore, the direct effect of the Cr and Mo segregation induced by the transformation of austenite was the primary factor governing the precipitation of the secondary phases.

**Figure 5:**
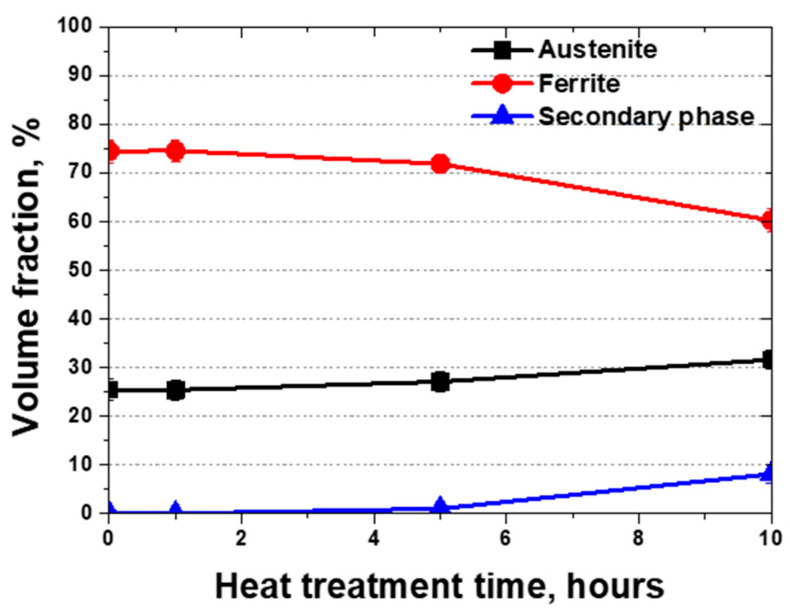
Heat treatment time vs. volume fraction curve for the super duplex stainless steel AISI2507 at 700 °C.

Upon exposure to 700 °C, AISI2507 exhibited variations in the volume fraction, as shown in Figure 5 and Table 3. Following a 10 h exposure at 700 °C, the fraction of austenite increased from 25.5% to 31.6%, accompanied by the precipitation of secondary phases [20,21,32]. As the fractions of the austenite and secondary phases increased, the ferrite fraction decreased. In particular, the precipitation of secondary phases occurred as the austenite expanded, resulting in the transformation of ferrite and a subsequent decrease in its volume fraction.

The heat treatment of AISI2507 resulted in the emergence of austenite and secondary phases. The secondary phases precipitated at the grain boundaries of austenite owing to changes in the growth of austenite [42,44]. Even a slight variation in the volume fraction, as low as 1.7% (From 25.4% to 27.1%), caused precipitation (1.1%) of the secondary phases. These secondary phases, induced by the transformation of austenite, facilitated the segregation of Cr and Mo, resulting in the precipitation of sigma, followed by the growth of chi and CrN.

When considering the utilization of AISI2507 as a material for Li-ion battery casings, it is evident that the precipitation of secondary phases is feasible [9,12,45]. Secondary phase precipitation increases the sensitization of AISI2507 due to the presence of austenite and ferrite, and the low binding energy of secondary phases [20,25]. However, a secondary-phase precipitation of 1% requires a minimum of 5 h. It takes up to 10 h for the phase fraction to reach 8%. Therefore, employing AISI2507 as a material for battery casings holds promise for enhancing safety, given its capacity to accommodate the precipitation of secondary phases, which fortifies its structural safety.

### 3.2. Electrochemical Behavior

By analyzing the electrochemical behavior of materials, one can ascertain both their corrosion resistance and the state of their microstructural composition. In this study, the corrosion resistance of AISI2057 at 700 °C was investigated using an electrochemical analysis. The analytical techniques employed include OCP measurements, EIS, and potentiodynamic polarization tests.

**Figure 6:**
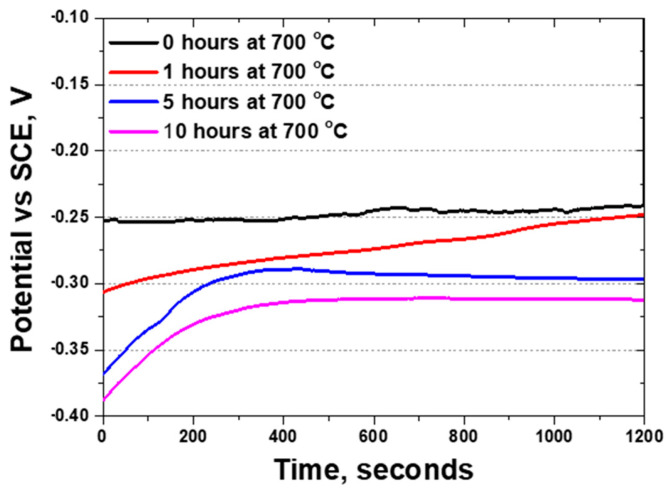
Time vs. potential curve. OCP results with heat treatment duration of super duplex stainless steel AISI2507 in 3.5 wt% NaCl electrolyte solution at 700 °C.

Although the potentials of pure metals can be directly calculated, the potentials of alloys or surface-treated materials cannot be determined using calculations alone. Hence, by analyzing the reactivity of materials via the OCP, the onset of oxidation and reduction reactions can be compared. OCP measurements were conducted over varying durations of exposure to 700 °C (Figure 6). As the heat treatment duration increased, the potential tended to increase. However, following the precipitation of secondary phases, the potential decreased from −0.25 to −0.32 V. Before the precipitation of secondary phases, an imbalance in the fraction of austenite and ferrite resulted in a lower OCP, which was subsequently elevated by the growth of austenite [14,20,21]. However, the precipitation of secondary phases led to a decrease in the potential from −0.25 V to −0.32 V.

**Figure 7:**
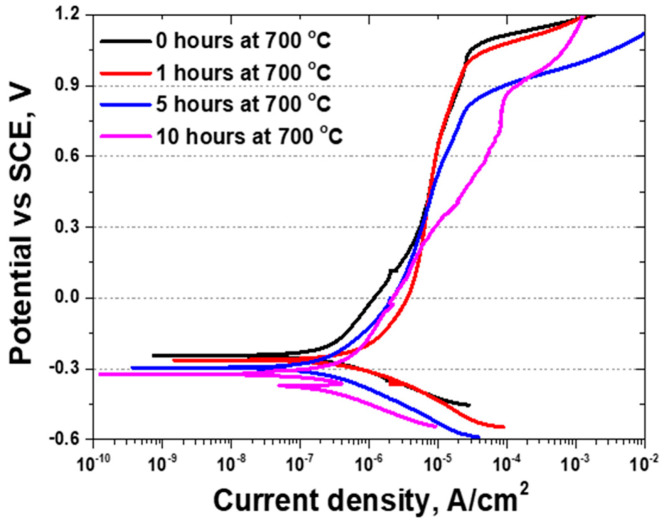
Potential (V) vs. current density (A/cm^2^) curve. Potentiodynamic polarization curve of super duplex stainless steel AISI2507 with heat treatment time at 700 °C in 3.5 wt% NaCl electrolyte solution.

The results of the potentiodynamic polarization test, which evaluates the variation in current density with potential, are shown in Figure 7. This test revealed that, at the active polarization curve of the potentiodynamic polarization curve, the current density (I_corr_) increased from 8 × 10^−6^ A/cm^2^ to 3 × 10^−6^ A/cm^2^ as the potential decreased from −0.6 V to 1.2 V. Despite the potential increase, the current density remained stable owing to a passivation layer, and a significant increase in the current density was observed beyond the corrosion potential (E_corr_). This corrosion behavior is characteristic of stainless steel, with SDSS exhibiting a high potential (E_pit_, pitting potential, breaking passivation layer). The influence of secondary phases was investigated upon exposure to 700 °C for 10 h, exhibiting an increase in the volume fraction of secondary phases up to 8.1%, which resulted in a double activation polarization curve being attributed to these phases [25,40].

The secondary phases induced various changes in the potentiodynamic polarization curves. The sigma and chi phases, formed by the secondary phases owing to differences in the Cr composition of austenite and ferrite, exhibited varying potentials for oxidation and reduction, contributing to the formation of a double layer during activation polarization. These sigma and chi phases can accelerate galvanic corrosion with decreasing potential (E_corr_), thereby increasing the uniform corrosion rate (Increased current density after active polarization, I_corr_) and lowering the breakdown potential. The electrochemical behavior of the secondary phase fractions at 1.1% and 8.1% differed, indicating that the effect of the secondary phases on electrochemical behavior varied with the volume fraction. When considering the application of SDSS as a battery case material, the decrease in corrosion resistance owing to the precipitation of secondary phases does not occur immediately but requires time, particularly when the fraction of secondary phases is between 1.1% and 8.1%.

**Figure 8:**
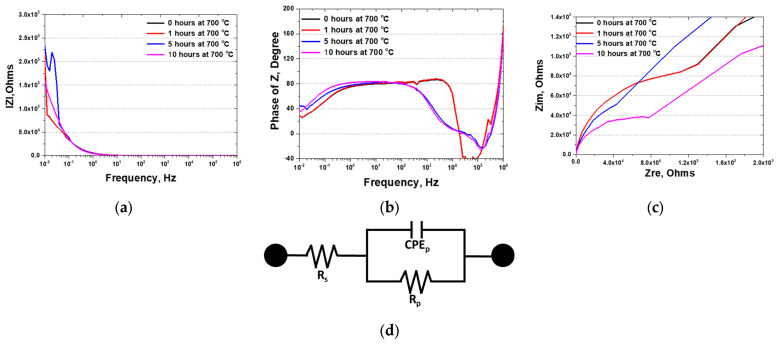
Electrochemical impedance spectroscopy from 10^6^ to 10^−2^ with a heat treatment time of super duplex stainless steel AISI2507 in 3.5 wt% NaCl electrolyte solution at 700 °C; (**a**) phase of Z (Degree) vs. Frequency (Hz) curve, Nyquist plot, (**b**) IZI (Ohms) vs. frequency (Hz) curve, Nyquist plot, (**c**) Zim (Ohms) vs. Zre (Ohms) curve, Bode plot, and (**d**) EIS circuit with resistance change.

In this study, EIS was employed to measure the variations in resistance with frequency, providing insights into the differences in surface conditions through resistance disparities. The specimens subjected to 700 °C over 0 to 10 h were analyzed (Figure 8 and Table 4). Secondary phase precipitation significantly affected the Nyquist plot, which revealed changes in the phase angle with frequency. The precipitation of the secondary phase was associated with a rapid increase in the phase angle at 10^−2^ Hz, accompanied by a modest rise in resistance. The distinctions attributable to the secondary phase in the Nyquist plot were further elucidated in the Bode plot, indicating a general decrease in material resistance owing to the precipitation of the secondary phase. 

Within the EIS results, the resistance of SDSS reflects the resistance (Figure 8c) of both the passivation layer (EIS circuit, Figure 8d) and the substrate material. An increase in the austenite fraction is correlated with elevated resistance, whereas secondary phase precipitation results in diminished resistance [18,46]. Augmentation of the austenite volume fraction by reducing the ferritization microstructure strengthens the passive layer. Conversely, the secondary phase affects the composition of critical elements, such as Cr and Mo, within the passivation layer, thus reducing its resistance from 82 kOhms to 39 kOhms. The influence of the secondary phase on the passivation layer is a critical factor influencing the corrosion resistance of the material, highlighting the intricate relationship between microstructural changes and electrochemical behavior in the context of high temperature exposure.

### 3.3. Discussion

In this study, we explored the application of AISI2507 duplex stainless steel as a potential material for Li-ion battery casings, specifically examining its microstructural and electrochemical behavior at elevated temperatures of 700 °C. Upon exposure to these temperatures, the material underwent a phase transformation from ferrite to austenite. As the duration of the exposure increased, the transformation progressed, with the fraction of austenite increasing by 5.5% (from 25.6% to 31.1%) and precipitation of the secondary phase increasing by 8.1% (from 0.0% to 8.1%). This secondary phase, which precipitated at the grain boundaries of austenite, consisted of the sigma, chi, and CrN phases [20,24].

The precipitation of these secondary phases considerably influenced the electrochemical behavior of AISI2507, altering the OCP. Following precipitation, the increase in the volume fraction of the secondary phase was correlated with accelerated galvanic corrosion rates, contributing to the formation of a double layer, as evidenced by the activation polarization of the potentiodynamic polarization curves. This phenomenon indicates that, even within the same material, different phases can exhibit distinct electrochemical properties, as demonstrated by the potentiodynamic polarization curves. In EIS studies, variations in resistance are indicative of the performance of the passivation layer, with a lower resistance suggesting a weaker passivation layer [17,31,47]. The emergence of secondary phases decreased the resistance, thereby weakening the passivation layer and diminishing the corrosion resistance.

Assuming the application of AISI2507 in Li-ion battery casings and its exposure to ignition conditions at 700 °C, it is observed that the precipitation of secondary phases decreases the strength, ductility, and corrosion resistance of the material. However, the precipitation of these detrimental secondary phases requires prolonged exposure, exceeding 5 h. Consequently, the utilization of AISI2507 for Li-ion battery casings suggests minimal performance degradation in the event of high temperature ignition due to the prevention of a decrease in strength due to the secondary phase, positioning AISI2507 as a viable material for enhancing the safety of Li-ion batteries.

## 4. Conclusions

To improve the safety of Li-ion batteries, AISI2507 was applied to Li-ion batteries, and the changes in the microstructure and electrochemical behavior in the event of ignition were analyzed, leading to the following conclusions:

(1) AISI2507, despite being a material with excellent strength and corrosion resistance at high temperatures, reacts sensitively to temperature changes. When an Li-ion battery ignites, the temperature can rise up to 700 °C, at which the microstructure of AISI2507 exhibits growth at the grain boundaries of austenite (from 25.5% to 31.6%) and the precipitation of a secondary phase (from 0.0% to 8.1%).

(2) Precipitation of the secondary phase in AISI2507 results in a decrease in corrosion resistance. As the volume fraction of the secondary phase increases up to 8.1%, the characteristics of the secondary phase become evident. This phase decreases the potential from −0.25 V to −0.32 V in the OCP and the resistance to frequency from 82 kOhms to 39 kOhms in the EIS. In the potentiodynamic polarization curves, the secondary phase reveals a decrease in the potential (E_corr_) from −0.25 V to −0.32 V and an increase in the current density (I_corr_) from 8 × 10^−6^ A/cm^2^ to 3 × 10^−6^ A/cm^2^ in the active polarization curve, indicating that corrosion could occur more readily and at a faster rate. A decrease in the pitting potential (E_pit_) from 1.05 V to 0.89 V also occurs, demonstrating that precipitation of the secondary phase weakens the corrosion resistance of AISI2507.

(3) When subjected to an intense heat of 700 °C for extended periods, AISI2507 demonstrates vulnerability, as the emergence of a secondary phase compromises its excellent corrosion resistance. Although subjected to such extreme conditions, lasting only an hour or less, this formidable material maintains its durability, exhibiting no signs of secondary-phase precipitation or microstructural transformation. Thus, the deployment of AISI2507 as a material for Li-ion battery casings shows an elevated level of safety, providing a barrier against the destructive effects of thermally induced fires as long as the duration of exposure is brief.

## Figures and Tables

**Table 1:** Chemical composition of super duplex stainless steel AISI2507 characterized by ICP-MS.

Elements	C	N	Mn	Ni	Cr	Mo	Cu	Fe
Chemical composition	0.01	0.27	0.8	6.8	25.0	3.8	0.2	Bal

**Table 2:** Chemical composition of super duplex stainless steel AISI2507 after heat treatment.

Phase		Volume Fraction	Chemical Composition, wt%				Fe	PRE
			N	Ni	Cr	Mo		
(a)	Austenite	56 ± 1.8%	0.49	7.8 ± 0.6	23.1 ± 1.1	3.1 ± 0.3	Bal	41.2
	Ferrite	44 ± 1.8%	0.05	5.7 ± 0.6	26.7 ± 1.1	5.0 ± 0.3	Bal	44.0
(b)	Austenite	49 ± 0.8%	0.51	7.9 ± 0.8	23.3 ± 1.2	3.2 ± 0.4	Bal	42.0
	Ferrite	51 ± 0.8%	0.05	5.8 ± 0.8	26.6 ± 1.2	4.7 ± 0.4	Bal	42.9
(c)	Austenite	27 ± 2.4%	0.79	8.1 ± 0.9	24.3 ± 1.3	3.3 ± 0.5	Bal	47.8
	Ferrite	73 ± 2.4%	0.05	6.6 ± 0.9	25.0 ± 1.3	4.4 ± 0.5	Bal	40.3

**Table 3:** Volume fraction of super duplex stainless steel AISI2507 with heat treatment times from 0 h to 10 h.

Heat Treatment Time	0 h	1 h	5 h	10 h
Austenite	25.5 ± 2.3%	25.4 ± 2.1%	27.1 ± 2.0%	31.6 ± 1.9%
Ferrite	74.5 ± 2.3%	74.6 ± 2.1%	71.9 ± 2.0%	60.3 ± 2.2%
Secondary phase	0.0 ± 0.0%	0.0 ± 0.0%	1.0 ± 0.6%	8.1 ± 1.8%

**Table 4:** Major values of EIS result with a heat treatment time at 700 °C of super duplex stainless AISI2507.

Condition	R_s_ (ohms)	C of CPE	n of CPE	R_p_ (kOhms)
0 h at 700 °C	6.1	10.1 × 10^5^	0.82	82
1 h at 700 °C	6.1	10.0 × 10^5^	0.82	82
5 h at 700 °C	6.1	6.3 × 10^5^	0.82	51
10 h at 700 °C	6.1	4.8 × 10^5^	0.82	39

## Data Availability

The original contributions presented in the study are included in the article, further inquiries can be directed at the corresponding authors.
